# Feasibility of Field Oral Health Assessment in Free‐Ranging Rhesus Macaques (Macaca mulatta) on Cayo Santiago

**DOI:** 10.1002/ajp.70210

**Published:** 2026-10-05

**Authors:** Jonathan Y. An, Johnny Hudson, Andrew Nordlie, Ryan O'Donnell, Josué E. Negrón‐Del Valle, Daniel Phillips, Nahiri Rivera, Angelina Ruiz Lambides, Noah Snyder‐Mackler

**Affiliations:** ^1^ Department of Oral Health Sciences, School of Dentistry University of Washington Seattle Washington USA; ^2^ Department of Periodontics, School of Dentistry University of Washington Seattle Washington USA; ^3^ Center for Evolution and Medicine Arizona State University Tempe Arizona USA; ^4^ Caribbean Primate Research Center University of Puerto Rico San Juan Puerto Rico USA; ^5^ School of Life Sciences Arizona State University Tempe Arizona USA; ^6^ School of Human Evolution and Social Change Arizona State University Tempe Arizona USA; ^7^ ASU‐Banner Neurodegenerative Disease Research Center Arizona State University Tempe Arizona USA

**Keywords:** aging, Cayo Santiago, field primatology, oral biospecimens, periodontal disease, rhesus macaque

## Abstract

Free‐ranging rhesus macaques (Macaca mulatta) on Cayo Santiago provide a uniquely characterized translational model for periodontal disease, supported by decades of demographic and genetic data. To assess the feasibility of conducting comprehensive periodontal examinations, oral biospecimen collection, and portable intraoral radiography in free‐ranging macaques during a 5‐day pilot field campaign. Eighteen macaques (7 males, 11 females; ages 9–23 years) were evaluated under ketamine/xylazine anesthesia. Periodontal charting (probing depth, clinical attachment level, bleeding on probing), multimodal biospecimen collection (gingival crevicular fluid, tongue swabs, supragingival plaque, whole saliva), and portable intraoral radiography (KaVo NOMAD Pro 2) were performed within an existing multi‐station sedation workflow. Full periodontal assessments were completed in 18/22 animals (82%). Mean probing depths were 2.0–2.2 mm, with significantly greater depths in aged animals (≥ 15 years; *p* < 0.05). GCF RNA and protein yields were inadequate for planned analyses. Tongue swabs from 15 animals yielded sufficient protein for 37‐analyte multiplex profiling; 31 analytes met the minimum criterion for age‐group comparisons. Exploratory analysis identified higher CD40L and IL‐8/CXCL8 in aged animals and modest difference in TNF‐α. Age‐by‐sex interactions were identified for IL‐8/CXCL8 and IP‐10/CXCL10. Radiographs showed age‐related alveolar bone loss without caries. Comprehensive oral health assessment is feasible in this field setting. Earlier dental‐station placement and improved GCF collection are needed. Tongue swabs offer a practical specimen for inflammatory profiling, supporting future longitudinal oral health phenotyping within the established Cayo Santiago infrastructure.

## Research Highlights

- Standardized periodontal examinations and oral biospecimen collection are feasible in free‐ranging rhesus macaques on Cayo Santiago.
- Tongue swabs yield sufficient protein for multiplex inflammatory profiling, whereas gingival crevicular fluid was insufficient under the pilot field protocol.
- Aged macaques had greater mean probing depth, and tongue‐swab analyses identified age‐group differences in selected inflammatory and repair‐associated mediators.

## Graphical abstract

A 5‐day field study on Cayo Santiago shows that standardized dental exams, oral sampling, and portable radiography can be performed in free‐ranging rhesus macaques. Tongue swabs yielded enough protein for inflammatory profiling, whereas gingival fluid samples did not, enabling scalable studies of oral health and aging.

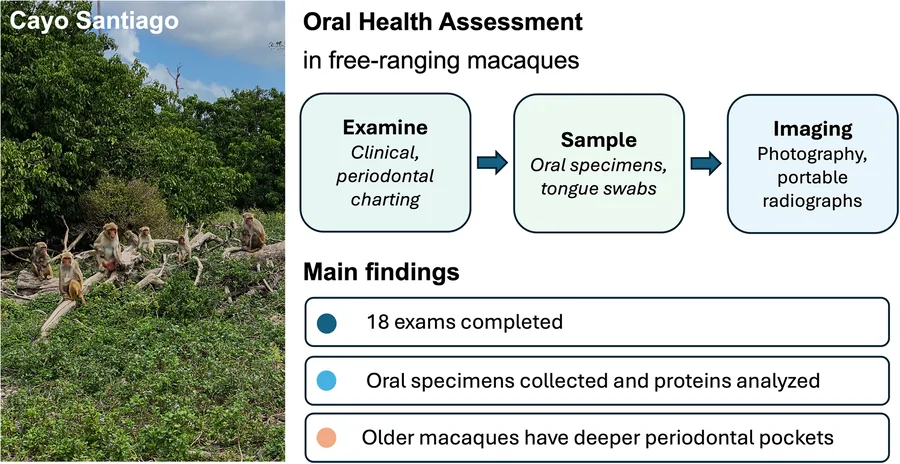

**Abbreviations**
AJP: American Journal of Primatology
ASP: American Society of Primatologists
BCA: Bicinchoninic acid assay
CPRC: Caribbean Primate Research Center
GCF: Gingival crevicular fluid
NHP: Non‐human primate
PD: Probing depth
SEM: Standard error of the mean
VEGF‐A: Vascular endothelial growth factor A

## 1 Introduction

Periodontal disease is a prevalent chronic oral inflammatory condition in humans, and its study benefits from animal models that closely mimic human oral biology. Non‐human primates (NHPs) are valuable models for oral health research because they share key similarities with humans in anatomy, immune responses, and microbiome composition (Chen et al. 2018; Ebersole et al. 2022; Kirakodu et al. 2019; Gonzalez et al. 2016). In particular, rhesus macaques (Macaca mulatta) naturally develop periodontal disease with clinical and microbiological features that resemble those in humans (Ebersole et al. 2022; Kirakodu et al. 2019; Gonzalez et al. 2016; Ebersole et al. 2025, 2016). Unlike rodent models that often require artificial induction of disease, NHPs can exhibit spontaneous periodontitis, especially as they age. These characteristics make NHPs well‐suited to studying age‐associated oral disease within a biologically relevant and translational framework.

Among available NHP populations, the free‐ranging rhesus macaque colony on Cayo Santiago offers a uniquely powerful setting to investigate oral health and disease in the context of natural aging. Cayo Santiago, a small island off the coast of Puerto Rico, is home to a free‐ranging colony of rhesus macaques that has been the focus of longitudinal research since 1938 (Newman et al. 2023; Turcotte et al. 2024a; Fernandes et al. 2023; Chiou et al. 2022). The colony was founded with 409 NHPs imported from India and has grown to over 1,000 monkeys (~1,500 animals in recent years). This population is managed by the Caribbean Primate Research Center (CPRC) with minimal human intervention beyond providing food and water, and there are no natural predators on the island. Uniquely, the Cayo Santiago rhesus macaque population has well‐documented maternal lineages over many decades, and genetic parentage analyses introduced in the late 20th century (1980s onward) have enabled reconstruction of extensive pedigrees across multiple generations (Chiou et al. 2020; Widdig et al. 2016), and life history has been recorded for decades, providing rich genetic and demographic data.

As the world's longest continuously monitored natural primate population, the Cayo Santiago colony offers a depth of longitudinal and environmental context rarely achievable in captive laboratory colonies and directly addresses limitations of traditional animal models of oral disease. Notably, the monkeys have experienced substantial environmental stressors, including the impacts of Hurricanes Maria in 2017 and Hurrican Fiona in 2022 which have been shown to influence social structure and physiological outcomes (Testard et al. 2021). Together, these features make Cayo Santiago an ideal naturalistic platform for studying oral health and periodontal disease across the aging trajectory, bridging the gap between controlled laboratory studies and human epidemiological research.

Despite extensive behavioral and epidemiological research on the Cayo Santiago macaques, no prior study has performed on‐site oral health examinations and oral sampling collection in this free‐ranging population. Previous oral health research in rhesus macaques has largely been limited to captive colonies or opportunistic sampling, which may not capture the full range of natural variation observed in free‐ranging populations. A recent review highlights the scientific value of the Cayo Santiago colony for studies of periodontal disease (Ebersole et al. 2025), demonstrating how this resource has been used to link disease outcomes with age, immune responses, and even heritability within matrilines. However, the practical feasibility of conducting in vivo oral health assessments or collecting biospecimens under true field conditions has not been tested.

Here, we report the first pilot study demonstrating the feasibility of conducting comprehensive oral health examinations and collecting oral samples from free‐ranging rhesus macaques on the remote island of Cayo Santiago. This report focuses on the logistical, methodological, and procedural considerations required to assess oral health in a field setting, including the successful execution of standardized periodontal examinations and the collection of multiple oral biospecimens. Our findings demonstrate that oral health assessments are feasible in this environment, establishing a foundation for future integrative studies of oral health, aging, and systemic biology in this uniquely characterized NHP population.

## 2 Methods

All study procedures were approved by the Institutional Animal Care and Use Committee (IACUC) of the University of Puerto Rico and collaborating institutions. This research adhered to the American Society of Primatologists (ASP) Principles for the Ethical Treatment of Non‐Human Primates and followed the ASP Code of Best Practices for Field Primatology. All procedures adhered to the legal requirements of the United States and the Commonwealth of Puerto Rico.

### 2.1 Study Population

The study was conducted at the Cayo Santiago Field Station in Puerto Rico, which oversees the rhesus macaque colony on Cayo Santiago Island. The island is approximately 15.2 hectares in area and lies ~1 km off the southeast coast of Puerto Rico. The macaques are free‐ranging and socially organized into naturally formed troops. All animals are habituated to human presence but are not routinely handled or medically examined. During the 5‐day project period, two trained dentists evaluated the oral health of rhesus macaques. All study procedures were approved by the University of Puerto Rico and collaborating institutions.

### 2.2 Data Collection

*Logistics and Trapping*. The oral health assessments were embedded within a multi‐station sedation workflow that included non‐dental evaluations (Figure 1). Oral examinations occurred late in the sequence, creating a feasibility constraint. A representative daily workflow included early departure to the island, morning trapping, sedation, and transport to the exam area, and data collection requiring about 20 min per animal before afternoon demobilization. These operational intervals were recorded as feasibility‐relevant timing, because station order and recovery timing directly determine completion probability.

**Figure 1:**
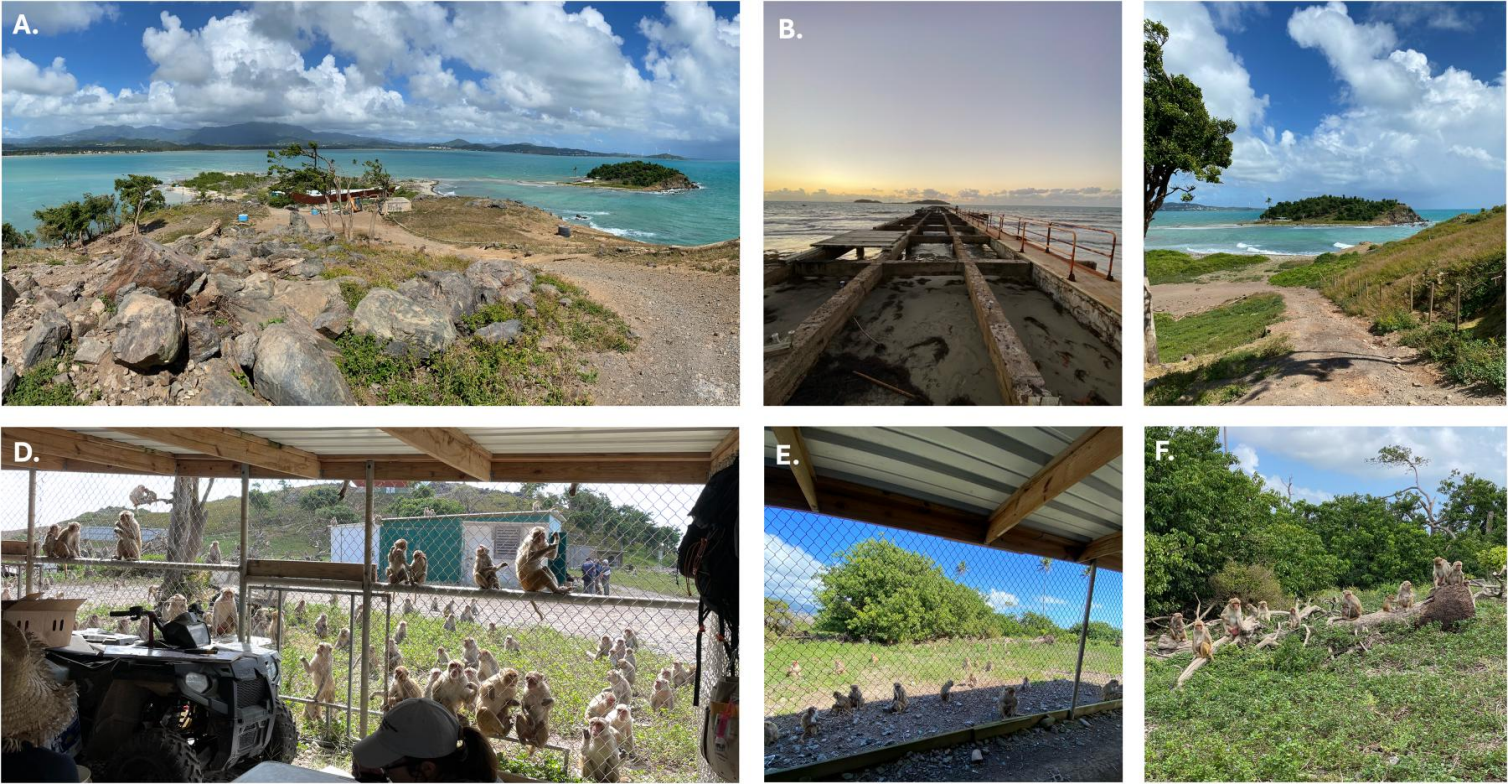
Representative images from Cayo Santiago, Puerto Rico, showing the island landscape, research infrastructure, and macaque observation areas. (A) Panoramic view of the rocky coastline, surrounding water, and nearby vegetated cay. (B) View along the dock/pier extending over the water. (C) Coastal trail and beach habitat with an offshore vegetated cay in the background. (D) Rhesus macaques gathered near a covered observation/work area separated by fencing. (E) View from within a sheltered observation area. (F) Macaques resting and moving through low coastal vegetation.

During the fall of 2024, animals were captured and anesthetized with intramuscular ketamine HCl (100 mg/mL solution; 1.0 mL for males and 0.8 mL for females) and intravenous xylazine (100 mg/mL solution; 0.05 mL for males and 0.03 mL for females). Additional ketamine and xylazine were administered intravenously as needed to sustain anesthesia. Upon arrival, animals were sedated according to established protocols. Morphometric measurements were collected first (Turcotte et al. 2024a, 2024b), followed by transthoracic echocardiography and a comprehensive ophthalmologic examination. Oral health assessments were performed last. Because dental examinations were performed at the end of the protocols, full oral assessments could not always be completed when animals began to recover from anesthesia.

*Oral Health Examination and Sample Collection*. While under anesthesia, each monkey underwent a comprehensive dental and periodontal examination modeled after standard human clinical protocols. Two dental examiners performed periodontal charting, with each tooth assessed at six sites using a periodontal probe. Periodontal probing depth (PD; mm) and gingival recession were recorded at each site to calculate clinical attachment level. Tooth‐level findings were documented, including missing or fractured teeth, visible plaque and calculus, gingival inflammation, suppuration, and tooth mobility.

The feasibility of collecting multiple oral biospecimens for downstream laboratory analyses was evaluated concurrently. Gingival crevicular fluid (GCF) was collected using sterile paper points placed within the gingival sulcus at designated sites per quadrant; strips were pooled and immediately immersed in RNAlater stabilization solution for RNA‐based analyses. Whole saliva was obtained by placing a Salivette® swab sublingually and then expressing it into a collection tube containing a protease inhibitor cocktail (ThermoFisher). Supragingival plaque was gently collected from molar surfaces using sterile curettes. Tongue swabs were collected by swabbing the dorsal surface of the tongue with sterile swabs to capture the mixed tongue‐associated proteome, including host epithelial and salivary proteins, microbial proteins, and antimicrobial peptides. All samples were kept as cold as possible during field collection and subsequently transported under cold conditions to the processing facility before shipment to the University of Washington for downstream analyses. Tongue‐swab samples were analyzed using a bead‐based multiplex panel targeting 37 cytokine, chemokine, and growth factor analytes (Procartaplex NHP Panel, Thermo Fisher Scientific).

*Portable Intraoral Radiography*. Intraoral dental radiographs were obtained in a subset of sedated animals using a handheld X‐ray system (Nomad Pro 2) with digital sensors. Radiographs were used to assess alveolar bone height, bone loss patterns, root morphology, retained root fragments, and other pathologies that may not be apparent by visual examination alone.

### 2.3 Data Analysis

Continuous variables are summarized as mean ± standard error of the mean (SEM), unless otherwise indicated; Luminex concentrations in the tables are summarized as mean (range). Animals were stratified into adult (< 15 years) and aged (≥ 15 years) groups based on the reported onset of age‐related survival decline in this population and the age distribution of the sampled cohort. Between‐group comparisons of periodontal pocket depth were performed using two‐tailed, unpaired Student's t‐tests in GraphPad Prism (v.11; GraphPad Software, San Diego, CA).

Protein concentrations were normalized to total protein as determined by BCA assay (Pierce). Sample were then analyzed using a bead‐based multiplex panel that quantified 37 cytokine/chemokine analytes (Procartaplex, NHP Panel, Thermofisher). For each Luminex analyte, an animal was considered detected when at least one of its two technical replicate wells yielded a numeric concentration. When both wells yielded numeric concentrations, their arithmetic mean was used; when only one well yielded a numeric concentration, that value was used. Animals with both wells reported as out of range below the assay range (OOR <) were excluded for that analyte. OOR < values were not assigned zero. Each animal contributed one independent observation, and the same animal‐level values were used for the age‐group comparisons, factorial analyses, and plots. Analyses used untransformed concentrations.

Adult and aged groups were compared using two‐sided, unpaired Welch's *t*‐tests when at least two detected animals were available in each group. Fold differences were calculated as the ratio of the aged to adult group means using unrounded values. Thirty‐one of the 37 analytes met the criterion for age‐group testing. Table 2 presents the 15 selected analytes. Supplemental Table 1 reports all 37 analytes, including results for all 31 eligible age‐group comparisons and descriptive results for the six analytes that did not meet the testing criterion.

Analytes with at least two detected animals in each of the four age‐by‐sex groups were evaluated using ordinary least‐squares two‐way ANOVA with age, sex, and their interaction as factors. Type III sums of squares were calculated using sum‐to‐zero contrasts; main effects compare equally weighted marginal means across the other factor. The full tongue‐swab cohort comprised three adult females, five adult males, five aged females, and two aged males, with analyte‐specific exclusions. Seven analytes met the factorial‐analysis criterion: CD40L, IFN‐γ, IL‐8/CXCL8, IL‐23, IP‐10/CXCL10, MCP‐1/CCL2, and VEGF‐A. Exploratory simple contrasts compared age groups within each sex and sexes within each age group using the full model's pooled residual variance and residual degrees of freedom (uncorrected Fisher's LSD approach). These four two‐sided contrasts were evaluated for each eligible analyte without an omnibus significance gate. Contrasts following nonsignificant interactions were not interpreted as evidence that age effects differed by sex. Factorial models assumed independent errors with common residual variance and approximately normal residuals.

We performed additional anlayses in R (R: A Language and Environment for Statistical Computing 2025; RCoreTeam 2017; Wickham et al. 2019), and with Python 3.12.14 using NumPy 2.3.5 and SciPy 1.17.0. All age‐group tests, factorial tests, and simple contrasts were exploratory. Given the exploratory and hypothesis‐generating nature of this pilot study, no correction for multiple comparisons was applied; all reported p‐values should therefore be interpreted with caution and treated as preliminary.

## 3 Results

### 3.1 Logistical Feasibility

During the 5‐day pilot period, the team conducted full oral health examinations on 18 macaques (7 males, 11 females; ages 9–23 years, Table 1). The field workflow successfully supported standardized periodontal charting and collection of multiple oral biospecimens when animals reached the dental station with adequate anesthetic depth (Figure 1). There were an additional four animals that could not be evaluated because oral assessments were conducted last in the multi‐station exam sequence, and some animals had begun to recover before the exam was completed.

**Table 1:** Demographics of NHPs Evaluated.

Sex	Males	Females
**Number**	*N* = 7	*N = 11*
**Age, Years, Mean ± SD**	12.9 ± 3.0 y/o	16.4 ± 4.3 y/o

The examination protocol used a Styrofoam bite block to facilitate intraoral access, followed by a general oral assessment that included a total tooth count, visual inspection for fractures, mobility, attrition, and any overt signs of oral pathology or infection (Figure 2). Full periodontal charting was completed in 18 animals, including probing depth, bleeding on probing, and clinical attachment loss. To standardize the procedure and minimize duration under sedation, oral biospecimens were collected only from the lower right quadrant. Gingival crevicular fluid was sampled using sterile paper points placed around both clinically healthy and diseased teeth. Supragingival plaque was collected with curettes, while tongue biofilm was sampled using sterile cotton swabs. Unstimulated whole saliva was pooled into cryotubes for downstream analysis.

**Figure 2:**
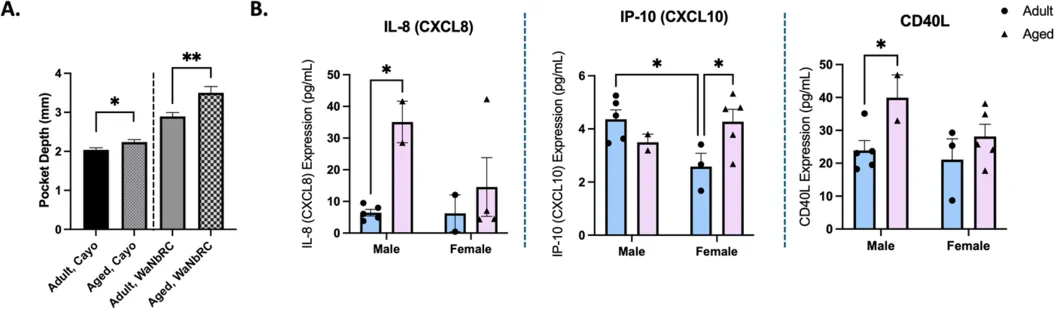
Clinical Photographs Demonstrating Periodontal Disease and Dental Collections. (A and D) Lateral intraoral view of an aged animal showing heavy staining with supragingival calculus. A close‐up of the dentition shows generalized gingival inflammation and recession, as well as compromised tooth structure. Mandibular anterior region demonstrating a paper point to collect GCF/plaque. (B) Dorsal tongue specimen collection and Styrofoam bite block. With retraction illustrating extensive plaque, staining, and calculus deposits (C). Periodontal probing highlights the extent of the pocket depth and soft tissue loss (D). (E) Maxillary anterior region showing attachment loss with root exposure, heavy staining, and calculus accumulation.

To supplement clinical assessments, portable dental radiography was piloted in the field using a KaVo NOMAD Pro 2 handheld x‐ray unit paired with digital sensors. Although only partially completed due to time constraints, this initial deployment demonstrated the feasibility of capturing diagnostic dental radiographs in a field environment. Taken together, these procedures affirm the viability of conducting comprehensive clinical and diagnostic oral health assessments in a free‐ranging NHP population under real‐world field conditions.

### 3.2 Periodontal Examination Findings

The macaques examined during the study ranged in age from 9 to 23 years, representing mid to late adulthood. Animals were categorized as adult (< 15 years) or aged (≥ 15 years), reflecting the onset of age‐related survival decline reported in free‐ranging rhesus macaque populations (Newman et al. 2023) and aligning with the upper range of ages observed in our cohort. All animals exhibited at least mild clinical signs of periodontal disease, with severity generally increasing with age. The average periodontal pocket depth across all subjects was approximately 2.0–2.2 mm (Figure 3). Common clinical findings included generalized calculus accumulation, heavy dental staining, gingival erythema, particularly in the molar regions, and evidence of tooth wear or fractures. Despite these indicators of early‐to‐moderate periodontal pathology, no animals showed overt abscesses or signs of purulent odontogenic infection. These preliminary findings suggest that free‐ranging rhesus macaques on Cayo Santiago develop mild to moderate periodontal disease with age, mirroring the age‐associated trajectory observed in humans and captive NHP populations, though the severity appears lower in this naturalistic setting (Figure 3A).

### 3.3 Biospecimen Yield and Downstream Assay Feasibility

RNA and protein were extracted from gingival crevicular fluid (GCF) samples to evaluate the feasibility. Qubit‐based quantification indicated that GCF RNA/protein concentrations were below the thresholds required to run the planned assays, limiting immediate feasibility for GCF‐based cytokine/transcript workflows under current field sampling and handling conditions. In contrast, tongue‐swab protein extraction produced concentrations above quantification thresholds and was compatible with downstream immunoassay workflows. Multiplex inflammatory profiling of tongue‐swab protein was performed using a bead‐based panel that quantified 37 cytokine/chemokine analytes (ProcartaPlex, NHP Panel, Thermofisher). Proteins were normalized by BCA Assay (Pierce). Using animal‐level concentrations, aged animals had higher CD40L and IL‐8/CXCL8 concentrations than adult animals (Table 2). TNF‐α also showed a modest age‐group difference (*p* = 0.0430), although only two animals per group contributed measurements. Mean VEGF‐A concentration was lower in aged animals, but the difference was not statistically significant (*p* = 0.129). Across the full panel, 31 of 37 analytes met the minimum criterion of at least two detected animals per age group; six were reported descriptively only (Supplemental Table 1). These comparisons were exploratory and unadjusted for multiple comparisons.

In exploratory two‐way ANOVAs of seven eligible analytes, CD40L showed an age effect (*p* = 0.008) without a significant age‐by‐sex interaction (*p* = 0.233). IL‐8/CXCL8 showed age and sex effects (*p* = 0.0004 and *p* = 0.008, respectively) and an age‐by‐sex interaction (*p* = 0.021). IP‐10/CXCL10 also showed an age‐by‐sex interaction (*p* = 0.004), without significant age or sex main effects (*p* = 0.262 and *p* = 0.176, respectively).

Unadjusted comparisons within age and sex groups showed higher IL‐8/CXCL8 concentrations in aged versus adult males (*p* = 0.0004) and in aged males versus aged females (*p* = 0.002). IP‐10/CXCL10 concentrations were higher in aged versus adult females (*p* = 0.004) and in adult males versus adult females (*p* = 0.003). CD40L concentrations were higher in aged versus adult males (*p* = 0.012) and in aged males versus aged females (*p* = 0.04998); however, these comparisons do not establish that the age effect differed by sex because the interaction was not significant (Figure 3B).

IL‐23 and VEGF‐A showed sex effects (p = 0.036 and p = 0.032, respectively), with lower IL‐23 and higher VEGF‐A marginal means in males. Neither showed a significant age effect or interaction. IFN‐γ and MCP‐1/CCL2 showed no significant main effects or interactions. All factorial analyses and within‐group comparisons were exploratory and unadjusted for multiple comparisons; only two aged males contributed to each eligible factorial model.

### 3.4 Radiographic Findings

To complement the periodontal examinations, intraoral dental radiographs were obtained in a subset of animals using a portable KaVo NOMAD handheld X‐ray unit paired with digital intraoral sensors. This mobile radiographic setup was selected due to its portability and digital sensors that enable rapid image acquisition and immediate review in the field, minimizing radiation exposure. Radiographs provide critical information not detectable through clinical examination alone, including assessment of alveolar bone height, periodontal bone loss patterns, root morphology, retained root fragments, and pathology. Radiographic analysis showed age‐related alveolar bone loss (Figure 4) and interproximal calculus in older animals. No cavities were noted. Furthermore, multiple animals had broken teeth, which did not lead to pathosis in their radiographs, other than retained tooth fragments.

**Figure 3:**
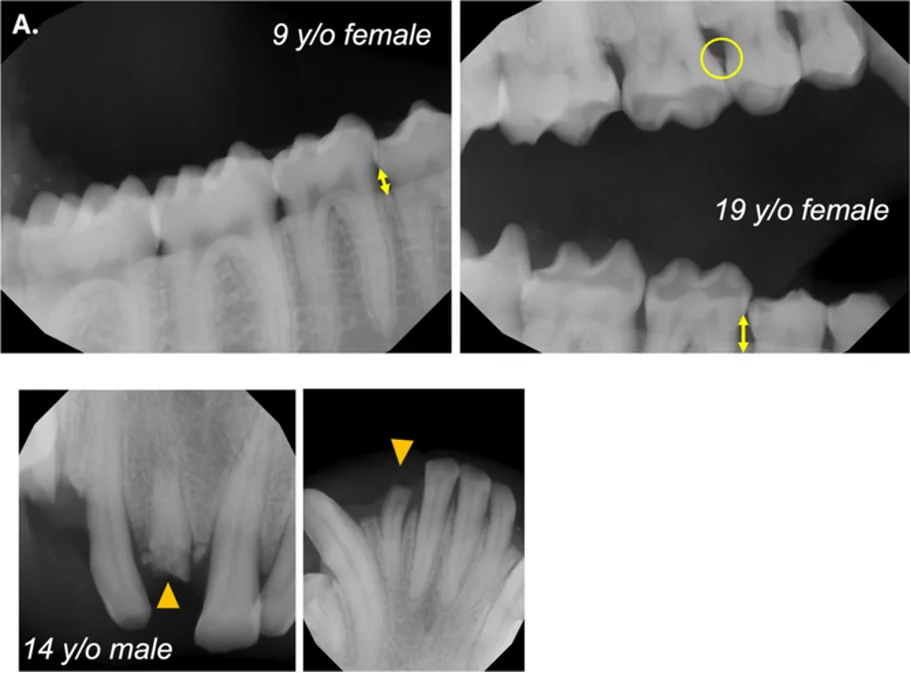
Aging is Associated with Increased Pocket Depth and Altered Inflammatory Marker Expression. (A) Mean mandibular probing depth for adult (< 15 years) and aged (≥ 15 years) Cayo Santiago macaques, shown alongside comparison data from the Washington National Biomedical Research Center (WaNBRC). Bars show mean ± SEM. *p < 0.05; **p < 0.01. (B) Tongue‐swab concentrations (pg/mL) of IL‐8 (CXCL8), CD40L, and IP‐10/CXCL10 stratified by sex and age group. Each symbol represents one animal; circles indicate adults and triangles indicate aged animals. Bars show mean ± SEM. Each analyte includes five adult males, two aged males, three adult females, and five aged females. Animal‐level concentrations were calculated as described in Methods. Brackets indicate comparisons of age groups within each sex and sexes within each age group, calculated using the full age‐by‐sex ANOVA model's pooled residual variance and residual degrees of freedom (uncorrected Fisher's LSD approach). The CD40L interaction was not significant, so its individual comparisons do not establish that the age effect differed by sex. *p < 0.05; **p < 0.01; ***p < 0.001. All *p*‐values are unadjusted for multiple comparisons.

**Figure 4:**
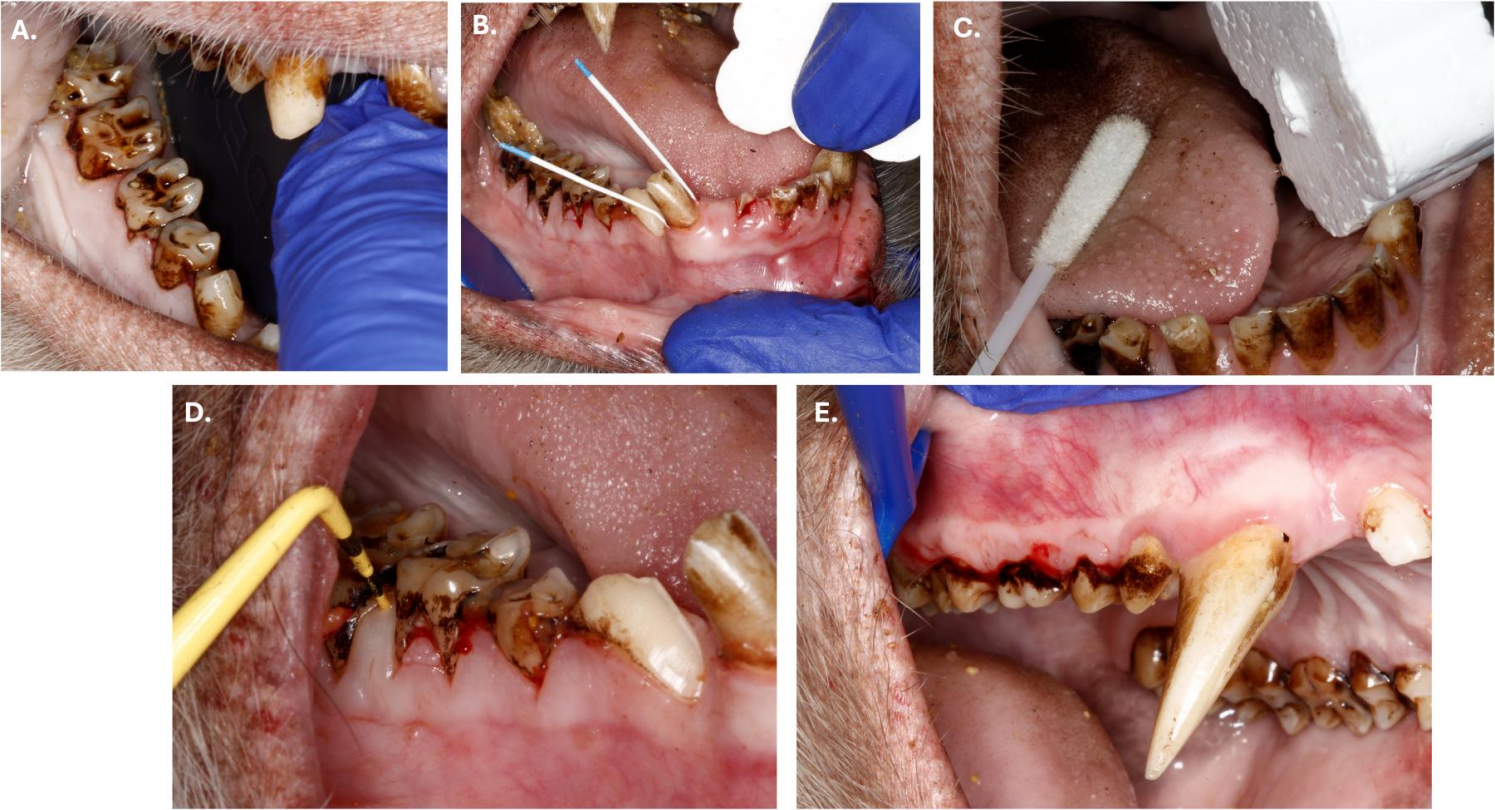
Representative Dental Radiographs Illustrating Age‐related and Structural Findings. (A) Bitewing radiographs from a 9‐year‐old female (left) and a 19‐year‐old female (right). The older individual demonstrates a relatively smaller pulp chamber and more radiographic evidence of alveolar bone loss (yellow arrows) than the younger individual. Radiopaque deposits consistent with dental calculus are also visible in the 19‐year‐old (yellow circle). (B) Periapical radiographs from a 14‐year‐old male demonstrating fractured teeth (orange arrowheads) without radiographic evidence of periapical pathology or other significant abnormalities.

## 4 Discussion

This pilot study demonstrates that comprehensive periodontal examinations and multimodal oral biospecimen collection can be conducted in a free‐ranging rhesus population on Cayo Santiago. These pilot data provide initial proof of concept for integrating oral health phenotyping into existing long‐term primate field protocols, contingent on workflow optimization. The site's unique monitoring infrastructure and demographic depth create a strong scientific rationale for adding standardized oral health endpoints, enabling longitudinal study designs that link oral inflammation to aging and ecology.

A major bottleneck was not the oral exam itself but the interaction between station order and anesthetic recovery. Because the dental station was scheduled last in a multi‐station protocol, some animals had begun to recover before oral assessments could be completed. This is a practical but scientifically consequential constraint: incomplete exams can introduce selection bias (those with longer/shorter recoveries or earlier station arrival) and reduce comparability across days. A second key feasibility outcome concerns the usability of biospecimens. Although GCF is an attractive matrix for local periodontal biomarkers, established paper point/Periopaper workflows highlight that GCF volumes are small and require careful isolation, defined sampling durations, and contamination control to protect analyte yield and interpretability. In this pilot, GCF RNA/protein yields were insufficient for planned assays, while tongue swabs produced adequate protein concentrations for multiplex immune profiling. This suggests that when workflow time is constrained, tongue swabs may provide a more robust, field‐ready inflammatory readout, while GCF collection may require protocol refinements (i.e., increasing the number of pooled strips, offering immediate snap‐freeze options, and/or reordering stations to enable earlier GCF collection).

Biologically, the dorsal tongue is a stable, highly vascularized mucosal surface that integrates host epithelial turnover, salivary secretions, microbial biofilm communities, and immune‐derived proteins. Tongue‐swab measurements may reflect a mixture of local epithelial, salivary, microbial, and immune‐associated sources. This study did not establish how these measurements relate to systemic inflammation or periodontal inflammation. The findings support tongue swabs as a practical specimen for exploratory protein profiling, but their value as markers of systemic immune aging remains to be determined. From a translational perspective, tongue sampling is minimally invasive, rapid, and field‐compatible. It does not require precise sulcular isolation, extended sampling time, or strict fluid volume thresholds. In constrained sedation workflows, this robustness is critical. The ability to profile a 37‐analyte panel from tongue swabs, with 31 analytes meeting the minimum sample‐size criterion for exploratory age‐group comparisons, supports the feasibility of immunologic profiling when local periodontal biospecimen yield is limited. Nominal age‐group differences in CD40L and IL‐8/CXCL8 support further investigation of tongue‐associated inflammatory proteins. The TNF‐α finding was based on only two detected animals per group and requires particular caution. Exploratory interactions for IL‐8/CXCL8 and IP‐10/CXCL10 suggest that age‐associated patterns may differ by sex, but the very small age‐by‐sex groups and absence of multiplicity correction preclude firm conclusions. These findings support larger, balanced studies and do not establish validated biomarkers of aging.

Portable intraoral radiography was feasible and valuable even when only partly completed. Published evaluations indicate that handheld systems can deliver image quality comparable to that of wall‐mounted units and that operator radiation exposure does not increase when operated by trained personnel according to manufacturer specifications (Nitschke et al. 2021). For field primate work, radiography adds value by validating alveolar bone loss patterns and identifying retained root fragments ‐ findings that can be missed with visual inspection alone, and that may be especially relevant in animals with fractures and heavy attrition. Ethically, invasive field procedures must be justified by scientific value and designed to minimize distress and risk. This study's reliance on existing veterinary sedation workflows and its explicit stopping logic when animals begin to recover are consistent with the American Society of Primatologists' principles, which emphasize the minimization of pain and distress and stewardship responsibilities in primate research. Future iterations would benefit from a tiered oral assessment design (brief screening vs full charting) and earlier placement of oral sampling in the station order to reduce non‐completion and maximize sample quality.

## 5 Limitations

The pilot sample size was modest and may not be representative of the broader colony distribution. Quadrant‐only biospecimen sampling improves time control but may miss spatial heterogeneity in periodontal disease. Radiography was performed on a subset and was time‐constrained. Biomarker analyses were exploratory and potentially confounded by age‐sex structure and small group sizes, and should be interpreted as evidence of feasibility. Tongue‐swab analyses were further restricted to the 15 of 18 examined animals that had sufficient protein yield; the three excluded animals were female. Analyte‐specific sample sizes varied because some animals had no numeric replicate readings. Animals with only one numeric replicate were retained using that reading, whereas animals with two numeric replicates were represented by their mean. Concentration comparisons therefore describe detected animals and may be affected by differences in detectability. Some age‐group comparisons relied on only two animals per group, and only two aged males contributed to each eligible factorial model. The small groups limited assessment of distributional assumptions and made interaction and simple‐contrast findings particularly uncertain. All *p*‐values were unadjusted for multiple comparisons; these findings require replication in a larger, more balanced cohort. Several modifications to address these limitations may be feasible within the existing field workflow. For example, relocating the dental collection station earlier in the sedation sequence, increasing the number of sites for the gingival crevicular fluid, and potentially adding immediate on‐site snap‐freezing. Other modifications require further methodological development before implementation at scale, including whole‐mouth rather than single‐quadrant sampling without extending sedation time, and standardizing portable radiography across operators and animals.

## 6 Conclusions

Standardized periodontal examinations, quadrant‐based oral biospecimen collection, and portable intraoral radiography are feasible in a free‐ranging rhesus field setting. The dominant constraints are workflow‐driven (station order and recovery timing) and matrix‐driven (low‐yield GCF RNA/protein under current procedures). Tongue swabs are a practical, higher‐yield alternative for multiplex inflammatory profiling under pilot conditions, while improved GCF protocols and earlier oral‐station scheduling are key next steps for enabling large‐scale assessment of local periodontal biomarkers (Table 2).

**Table 2:** Adult vs Aged Detection Frequency of Cytokine, Chemokine, and Growth Factor with Concentrations.

Analyte	Detected adult animals (of 8)	Detected aged animals (of 7)	Adult mean (range), pg/mL	Aged mean (range), pg/mL	Aged/adult ratio	p‐value	Significance
CD40L	8	7	22.861 (16.270–35.105)	31.518 (22.335–46.865)	1.38	0.0417	*
IL‐8 (CXCL8)	8	7	5.695 (0.380–9.470)	19.233 (4.760–41.630)	3.38	0.0419	*
TNF‐α	2	2	1.025 (0.880–1.170)	1.940 (1.830–2.050)	1.89	0.0430	*
VEGF‐A	8	7	616.763 (337.860–1001.585)	399.751 (259.745–982.330)	0.65	0.1292	*n.s*.
IFN‐γ	7	7	2.176 (1.295–3.195)	2.549 (1.155–3.610)	1.17	0.3815	*n.s*.
MCP‐1 (CCL2)	8	7	2.464 (1.035–7.945)	1.653 (0.750–2.450)	0.67	0.3640	*n.s*.
IL‐18	6	4	11.524 (2.580–54.765)	4.414 (3.240–6.140)	0.38	0.4492	*n.s*.
IL‐10	4	5	0.311 (0.240–0.440)	0.841 (0.190–2.430)	2.70	0.2908	*n.s*.
G‐CSF (CSF‐3)	3	5	4.290 (3.080–5.210)	2.539 (1.660–3.565)	0.59	0.0875	*n.s*.
VEGF‐D	6	6	5.612 (0.605–16.790)	3.051 (0.845–7.350)	0.54	0.3822	*n.s*.
IP‐10 (CXCL10)	8	7	3.691 (1.665–5.250)	4.046 (3.190–4.805)	1.10	0.4556	*n.s*.
I‐TAC (CXCL11)	3	6	7.630 (2.020–14.600)	5.385 (1.190–7.265)	0.71	0.6096	*n.s*.
IL‐23	8	6	30.235 (8.675–63.980)	25.762 (2.250–53.105)	0.85	0.7267	*n.s*.
GM‐CSF	3	5	7.650 (5.570–11.245)	7.398 (6.155–9.120)	0.97	0.9039	*n.s*.
IL‐12p70	7	6	0.178 (0.100–0.215)	0.182 (0.030–0.350)	1.02	*0.9411*	n.s.

*Note:* Detection frequency is the number of animals with at least one numeric technical replicate reading. Concentrations are summarized as mean (range) in pg/mL across detected animals, using the mean of two numeric readings or the single numeric reading when only one was available. The aged/adult ratio is the aged group mean divided by the adult group mean. Age groups were compared using two‐sided, unpaired Welch's *t*‐tests when at least two detected animals were available in each group. *p < 0.05; n.s., not significant. All *p*‐values are exploratory and unadjusted for multiple comparisons.

## Supporting information


**Table S1: Detection frequency and concentrations of 37 analytes in tongue‐swab samples.** Tongue‐swab protein was sufficient for multiplex analyses in 15 of the 18 examined macaques. Counts indicate animals with at least one numeric technical replicate reading. Concentrations are animal‐level mean (range) in pg/mL, calculated as described in Methods. Fold difference is the aged/adult mean ratio. 31 analytes met the minimum criterion of at least two detected animals per age group and were compared using two‐sided, unpaired Welch's *t*‐tests; 6 are descriptive only. Shaded rows indicate analytes differing significantly between age groups; *p < 0.05; n.s., not significant. All *p*‐values are nominal and unadjusted for multiple comparisons. Dashes indicate unavailable concentration summaries or ratios, or comparisons not performed because the minimum sample‐size criterion was not met. A range is not reported when only one animal contributed a measurement.

## Data Availability

The data and analysis code supporting the findings of this study are available from the corresponding author upon reasonable request.
